# The Key Role of Lysosomal Protease Cathepsins in Viral Infections

**DOI:** 10.3390/ijms23169089

**Published:** 2022-08-13

**Authors:** Melania Scarcella, Danila d’Angelo, Mariangela Ciampa, Simona Tafuri, Luigi Avallone, Luigi Michele Pavone, Valeria De Pasquale

**Affiliations:** 1 Department of Molecular Medicine and Medical Biotechnology, Medical School, University of Naples Federico II, Via S. Pansini n. 5, 80131 Naples, Italy; 2 Department of Veterinary Medicine and Animal Productions, University of Naples Federico II, Via Delpino 1, 80137 Naples, Italy

**Keywords:** cathepsins, viruses, infection, physiopathology, therapy

## Abstract

Cathepsins encompass a family of lysosomal proteases that mediate protein degradation and turnover. Although mainly localized in the endolysosomal compartment, cathepsins are also found in the cytoplasm, nucleus, and extracellular space, where they are involved in cell signaling, extracellular matrix assembly/disassembly, and protein processing and trafficking through the plasma and nuclear membrane and between intracellular organelles. Ubiquitously expressed in the body, cathepsins play regulatory roles in a wide range of physiological processes including coagulation, hormone secretion, immune responses, and others. A dysregulation of cathepsin expression and/or activity has been associated with many human diseases, including cancer, diabetes, obesity, cardiovascular and inflammatory diseases, kidney dysfunctions, and neurodegenerative disorders, as well as infectious diseases. In viral infections, cathepsins may promote (1) activation of the viral attachment glycoproteins and entry of the virus into target cells; (2) antigen processing and presentation, enabling the virus to replicate in infected cells; (3) up-regulation and processing of heparanase that facilitates the release of viral progeny and the spread of infection; and (4) activation of cell death that may either favor viral clearance or assist viral propagation. In this review, we report the most relevant findings on the molecular mechanisms underlying cathepsin involvement in viral infection physiopathology, and we discuss the potential of cathepsin inhibitors for therapeutical applications in viral infectious diseases.

## 1. Classification, Synthesis, Cellular Localization, and Physiopathological Roles of Cathepsin

Cathepsins include a family of lysosomal protases, so-called from the Greek term kathepsein (to digest) to indicate proteases that are active in a slightly acidic environment [1,2,3]. Since the discovery of the first cathepsin in late 1920, to date, more than 20 types of cathepsins have been identified in all living organisms. In humans, cathepsins comprise 15 proteolytic enzymes that are structurally classified on the basis of their catalytic active site residue, namely serine (cathepsin A and G), aspartate (cathepsin D and E), or cysteine (cathepsin B, C, F, H, K, L, O, S, V, X, W, Z). Most of the cathepsins act as endopeptidases, although cathepsins A, B and X may also work as carboxypeptidases and cathepsin H operates as an aminopeptidase [4].

Almost all types of cathepsins are synthesized through a common pathway that starts in the ribosome, with the synthesis of a precursor molecule containing a signal peptide, a precursor peptide, and a catalytic domain. The precursor molecule translocates to the endoplasmic reticulum, where it undergoes the hydrolysis of the signal peptide and progresses to glycosylation. The protein is then transported to the Golgi apparatus where it is further glycosylated and phosphorylated to form a mannose-6-phosphate (M6P) protein, which is specifically recognized by M6P lysosomal receptors, ensuring its transport to the endosomal/lysosomal system [5]. However, some evidence demonstrates the existence of alternative routes for the intracellular transport of the newly synthesized procathepsins that involve lysosomal integral membrane protein (LIMP-2) and sortilin [6]. In the lysosome, the hydrolysis of the precursor protein at a low pH leads to the removal of the prodomain yielding active and mature cathepsin.

The maturation process of procathepsins may occur through either auto processing and self-activation or by other protease catalysis, or through both modes [7,8,9,10,11,12,13,14]. While cathepsins B, H, K, L, and S undergo autoactivation, cathepsins C and X require cathepsins L and S for their activation [7]. On the other hand, cathepsin D maturation proceeds through partial autoactivation and activation by cathepsin B and L [14]. The autocatalytic activation is mediated by glycosaminoglycans (GAGs) [15,16,17], linear negatively charged polysaccharides present in the lysosomes as well as on the cell surface and extracellular matrix (ECM) where they regulate important processes in development, homeostasis, and disease [18,19,20,21]. Procathepsin—GAG interaction triggers a conformational change in the precursor molecule that facilitates processing by another procathepsin molecule [10,16,17,22].

In the endolysosomal compartment, cathepsins carry out the proteolytic processes needed to degrade the cargo transported to the endolysosomes, thus contributing to the protein turnover and the normal metabolism of the cell. In this compartment, cathepsins play a pivotal role in autophagy, regulating the biogenesis and the cellular population of lysosomes and autophagosomes as well as the autophagic flux [8,23,24,25,26]. The involvement of cathepsins in the autophagic pathway is relevant in light of the fundamental role of such a process in neuronal development and degeneration [27,28,29,30]. In addition, cathepsins have been shown to regulate growth and development-related processes through their hydrolytic effect on various hormones and growth factors [3,8,22,28,29,30,31]. In the endosomes of immune cells, they participate in both the innate and adaptative immune responses [32,33,34,35,36]. Deregulation of the expression or activity of cathepsins in the endolysosomes leads to impaired degradation of organelle cargos, resulting in the accumulation of substrates that may be responsible for various pathological conditions [3,8,37,38,39,40,41,42,43,44]. These conditions include lysosomal storage diseases (LSD) such as neuronal ceroid lipofuscinosis [37]; galactosidases [38]; mucopolysaccharidoses [39] and Gaucher disease [40]; Alzheimer’s [41,42]; Parkinson’s and Huntington’s diseases [43]; type I diabetes [44]; auto-immune diseases [3]; and others [8].

**Figure 1:**
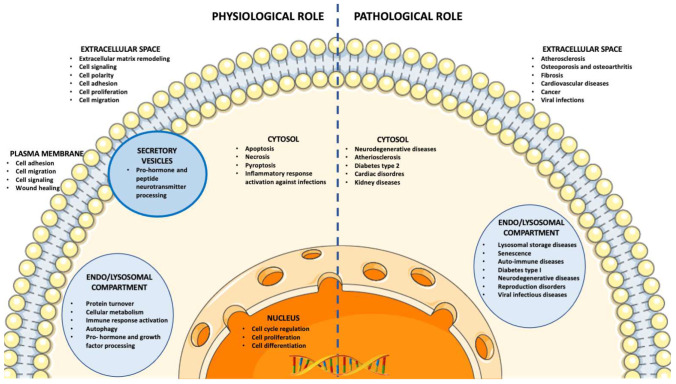
Physiological and pathological roles of cathepsins depending on their localization at a specific cellular compartment.

Although cathepsins are mainly located in the lysosomes, where they show the highest activity due to the low pH of these organelles, they are also active outside of the endolysosomal compartment (cytosol and extracellular space). It should also be noted that cathepsins can occur, bind, and are catalytic active on the cell surface. Indeed, they can be released in the cytosol through the lysosomal membrane permeabilization induced by a variety of stimuli, such as lysosomotropic agents, oxidative stress, and some cell death effectors [8,45]. In addition to lysosomal membrane permeabilization, other mechanisms may lead to extralysosomal translocation of cathepsins. These mechanisms, which include abnormalities in their biosynthetic machinery, generating cathepsins lacking the signal peptide or truncated cathepsins with modified signal sequences, may direct cathepsin variants to the cytosol as well as to the mitochondria or nucleus. In the cytosol, cathepsins regulate apoptosis by both activating apoptotic proteases and degrading antiapoptotic proteins [2,8,46,47], and mediate inflammatory responses by activating inflammasome [32,33,34,35]. Loss of function or inactivation of cathepsins in the cytosol have been associated with pathological conditions such as neurodegenerative diseases, atherosclerosis [48,49], type 2 diabetes [50,51] kidney diseases [52,53], and ischemia [54,55,56]. In the nucleus, cathepsins are involved in processing transcription factors that regulate the cell cycle, cell proliferation, and differentiation, and therefore, dysregulation of nuclear cathepsins may contribute to the transformed phenotype of cancer cells [57]. Finally, cathepsins are secreted through lysosomal exocytosis or alternative trafficking routes in the extracellular milieu, where they participate in plasma membrane repair, bone remodeling, wound healing, and peptide prohormone processing [8,22]. Extracellular cathepsins are involved in the regulation of extracellular matrix (ECM) remodeling, which plays a fundamental role in the control of cell adhesion, proliferation, polarity, migration, and activation of cell signaling [58,59]. Therefore, cathepsins have been implicated in many diseases such as cancer, tissue fibrosis, osteoarthritis, and other pathological conditions associated with altered ECM homeostasis [8,22]. Figure 1 reports the different localization of cathepsins at distinct cellular compartments and their relative physiological and pathological roles.

## 2. Aid of Cathepsins to Viruses in the Host Cell Infection

Regardless of their cellular localization, cathepsins have been shown to play an important role in the host cell infection by various types of viruses [60]. Indeed, cathepsins can support the virus’s entry into the target cells, enable virus replication in the infected cells, and promote virus release and spread. Here, the involvement of cathepsins in the physiopathology of viral infections is reported.

Firstly, cathepsins have been shown to strongly affect the infection efficiency of many viruses by modulating their binding to host cell receptors and entry. Indeed, recognition and interaction with cellular receptors is a critical initial step of the viral cell cycle, regulating viral tissue tropism and pathogenesis [61]. The interaction with target cell receptors, which not only serves for attachment but also triggers viral entry and trafficking, is mediated by specific viral proteins expressed on the surface of both enveloped and non-enveloped viruses [62]. In many cases, viral attachment proteins require proteolytic activation by host cell proteases. Cathepsins B and L have been implicated in the proteolytic cleavage of the viral glycoprotein (GP) of the Ebola virus (EBOV) that facilitates virus interaction with the cellular receptor(s) and its entry into target cells [63,64,65]. Interestingly, faster viral fusion kinetics and enhanced infectivity of the Ebola strain named Makona, which carries an A-to-V substitution at position 82 (A82V) in the GP, have been correlated with a more efficient GP processing by cathepsin L [66]. Both cathepsins B and L seem to be also involved in the entry initial step of infection by human papillomavirus type 16 virus (HPV16) [67,68,69]. Furthermore, cathepsins B, L, and S mediating the disassembly of viral particles after endocytosis are required for reovirus entry [70,71,72]. Enzymatic activity of Cathepsin B and L is also utilized by severe acute respiratory syndrome (SARS) coronavirus (CoV) to infect cells expressing angiotensin-converting enzyme 2 (ACE2) receptor [73,74]. Indeed, CoVs, encompassing a large variety of viruses infecting many species of birds and mammals, including humans, employ a diverse array of entry strategies to infect target cells [75]. In particular, CoV entry may occur either via fusion directly at the cell surface or through an endocytic pathway. The spike surface envelope glycoprotein (S), which bears receptor binding and membrane fusion capabilities, is required for viral entry. The S protein is homotrimeric, with each subunit containing the S1 and S2 domains, the former mediating the host receptor binding and the latter required for fusing host and viral membranes [76]. Activation of S protein by proteolytic cleavage is required for viral entry into target cells: cell surface protease activity allows direct membrane fusion, whereas endosomal and lysosomal proteases are involved during endocytosis [77]. In addition to SARS-CoV, the involvement of cathepsins B and L has been demonstrated for Middle East respiratory syndrome coronavirus (MERS-CoV) and SARS-CoV-2, which may exploit both routes of entry depending on the host cells [74,78,79,80,81,82,83,84]. Besides cathepsins B and L, a role for cathepsins K, S, and V in SARS-CoV-2 entry into target cells has also been suggested [85]. Indeed, like other CoVs, to gain entry into target cells, SARS-CoV-2 depends on cleavage and activation of the S protein by host cell proteases that include furin, transmembrane protease serine 2 (TMPRSS2), and cathepsins [80,81,82,83,84,85]. Although the interplay between these host proteases during SARS-CoV-2 infection remains to be fully elucidated, amino acid sequences of the S protein that are susceptible to cleavage by cathepsins and that are highly conserved among all known SARS-CoV-2 variants have been identified [84,85]. In all regions of the spike protein, including the S1/S2 region critical for activation and viral entry, there are amino acid sequences susceptible to cleavage by cathepsins B, K, L, S, and V [85]. Cathepsins not only promote viral infection upon viral entry into target cells, but also activate viral fusion proteins at a late stage of replication. Indeed, cathepsin W activity is required for influenza A virus (IAV) entry at the stage of viral fusion in late endosomes [86]. Furthermore, cathepsins L and B play an important role in promoting the spread of highly pathogenic paramyxoviruses, such as Nipah and Hendra viruses, by converting the viral fusion protein to a mature and fusogenic form in the endosomal compartment [87,88].

Interestingly, cathepsins play a key role in promoting virus release and spread by upregulating and processing the host enzyme Heparanase (HPSE), an endoglycosidase that degrades the glycosaminoglycan heparan sulfate (HS) [89,90,91,92,93,94]. Human HPSE mRNA encodes for a 61.2-kDa protein containing 543 amino acids. Cathepsin L cleaves the proenzyme generating the active form consisting of 8 and 50 kDa subunits that associate noncovalently [95]. Active HPSE is responsible for the degradation of HS chains covalently attached to the extracellular matrix and plasma membrane core proteins forming HS proteoglycans (HSPGs), which are involved in a wide range of physiological functions [96,97,98]. Notably, HSPGs assist viruses in infecting target cells at various steps of their life cycle: they utilize HSPGs for attachment at the cell surface, entry, intracellular trafficking, egress, and spread [94,99,100,101]. Recent evidence demonstrates that host-encoded HPSE is upregulated and required for the release of viral progeny after herpes simplex virus 1 (HSV-1) and 2 (HSV-2) infection [88,90,102,103]. The removal of HS chains by HPSE facilitates the release of the newly made viral particles from the cells and their spread. During the productive phase of HSV-2 infection, the upregulation of HPSE correlated with increased levels of cathepsin L, and the inhibition of either HPSE or the cathepsin resulted to be detrimental to the infection [103]. Similar findings were reported for porcine reproductive and respiratory syndrome virus (PRRSV) whose infection causes upregulation of cathepsin L and heparanase, leading to a decrease of cell surface HS chains and, in turn, promoting viral release [104]. In addition, upregulation of cathepsin L and HPSE is involved in the pathogenesis of Dengue virus (DENV) infection [105,106]. Roles in the virus release and spread for cathepsins and HPSE have also been demonstrated in HPV16 [107], respiratory syncytial virus (RSV) [108], and hepatitis C virus (HCV) [109], as well as some CoVs and SARS-CoV2 infections [94,110,111,112].

A relevant aspect of cathepsin involvement in viral infections is their roles in antigen processing and presentation (host adaptative immune response) and activation of toll-like receptors (innate immune response) [113,114,115,116,117,118]. Indeed, cathepsins are known to degrade endocytosed and endogenous antigens to antigen peptides that bind to the major histocompatibility (MHC) class II molecules [113,114,115,116,119]. On the other hand, viruses exploit multiple mechanisms to evade immune recognition, including the manipulation of host antigen processing and presentation mechanisms [120]. This strategy to escape immune response enables the viruses to efficiently replicate in the infected cells. One example is provided by the ectromelia virus, which suppresses the expression of cathepsins B, L, and S in conventional dendritic cells to avoid host immune response and productively replicate [121]. Furthermore, cathepsins B, C, S, and Z were found to be downregulated in dendritic cells infected by human immunodeficiency virus type 1 (HIV-1), resulting in enhanced virus replication and transfer to contacting T lymphocytes, but decreased HIV-1 antigen processing and presentation to these T cells [122]. Increased levels of cathepsin B associated with impaired MHC class II antigen-processing pathways were found in IAV infection in vitro and in vivo [123]. Indeed, in IAV infection, cathepsin B has also been involved in progeny virion production [124]. By contrast, decreased expression levels of cathepsin S associated with an impairment of MHC class II maturation were observed in dendritic cells exposed to HCV or in hepatocytes expressing HCV proteins [125]. In SARS-CoV-infected monocytes, downregulation of the expression of cathepsins A, S, and H involved in antigen presentation and processing was found, suggesting a limited activation of a favorable adaptive immune response against this virus [126].

In addition, some viruses have developed strategies to evade the host innate immune response that involve the activation of various pattern recognition receptors (PRRs), including toll-like receptors (TLRs), among others, and the subsequent signaling resulting in the production of proinflammatory cytokines and/or the activation of programmed cell death [114,117,118,120,127,128,129]. For example, IAV is recognized by various PRRs, depending on the cellular compartment, the different types of cells, and the different stages of infection [130], and may also trigger PRR activation mechanisms to subvert the innate immune response [131,132]. Indeed, TRL activation leading to autophagy and apoptosis is subverted by IAV to enhance virion stability [133,134,135] and to facilitate its replication [136]. Activation of RIG-I-like receptors by RSV infection is associated with the overexpression of cathepsins B, C, E, G, H, K, L, S, W, and Z in infected mouse airways [118]. In HBV infection, impairment of autophagy correlated to an accumulation of immature lysosomes in infected cells has been demonstrated. The analyses of clinical specimens from chronic HBV-infected patients showed enhanced levels of cathepsin D in the liver tissues [137]. Cathepsin B acts as an upstream activator of the intrinsic apoptotic pathway that is exploited by noroviruses to expand the window time of their replication [138]. Both cathepsins B and S have been shown to contribute to apoptosis via caspase activation in DENV infection [139]. Furthermore, cathepsin B has been shown to exacerbate coxsackievirus B3-induced myocarditis in mice through activating inflammasome and promoting pyroptosis, a type of programmed cell death [140]. Interestingly, highly pathogenic human CoVs, including SARS-CoV, MERS-CoV, and SARS-CoV-2, besides suppressing interferon-mediated antiviral response, trigger massive cell death and cytopathy that release a large number of virion particles, thus facilitating viral dissemination [141]. In particular, transcriptomic analysis of peripheral blood mononuclear cells from COVID-19 patients demonstrated a remarkable increase of cathepsins B and L associated with the apoptotic pathway [142]. However, cathepsin activity may also contribute to the antiviral immune response by reducing viral replication. This is the case for cathepsin C, which has been shown to limit acute cytomegalovirus (CMV) infection in mice [143]. Table 1 summarizes the diverse roles of distinct cathepsins in the human infectious diseases caused by viruses.

## 3. Cathepsins as Potential Targets for Antiviral Therapies

Targeting cathepsins has proven to be a valid strategy for the development of effective antiviral drugs. A comprehensive list of cathepsin inhibitors is present in the MEROPS database (http://www.ebi.ac.uk/merops/, accessed on 20 July 2022) [144]. Furthermore, an elegant review by Pišlar and coworkers [74] reports an updated list of cathepsin inhibitors tested for Cov inhibition, including SARS-CoV-2, while a review by Liu and co-workers [145] nicely describes the antiviral properties, pharmacology, and toxicity of seven cathepsin L selective inhibitors that may represent an effective therapeutic option for COVID-19. Due to the diverse roles of cathepsins in promoting viral infections, different cathepsin-mediated pathways can be targeted to effectively fight the propagation and transmission of viruses. Herein, we report some examples of specific cathepsin inhibitors and their mechanisms of antiviral action.

In order to block cathepsin-mediated host cell entry and, in particular, the endosomal proteolysis step of entry, the cysteine protease inhibitor K11777, (2S)-N-[(1E,3S)-1-(benzenesulfonyl)-5-phenylpent-1-en-3-yl]-2-{[(E)-4-methylpiperazine-1-carbonyl] amino}-3-phenylpropanamide) and closely related vinylsulfones were developed, proving particularly effective for the treatment of filoviruses, such as EBOV and some paramyxoviruses [146]. The cysteine protease inhibitor K11777 has also been shown to inhibit CoV infection, but only in cell lines lacking activating serine proteases. In target cells expressing cell surface serine protease, only the use of both K11777 and a serine protease inhibitor such as camostat showed antiviral activity in an in vivo animal model of SARS-CoV infection [146]. The combined use of camostat or other serine protease inhibitors targeting TMPRSS2 and cathepsin inhibitor apilimod has been proven to strongly block SARS-CoV-2 infection in different cell types [147,148,149]. However, apilimod has been shown to dampen host immune response against SARS-CoV-2, leading to the exacerbation of the already impaired T cell immunity in affected patients, thus suggesting caution in its application [150]. Among the small molecules targeting cathepsins B and/or L, MDL 28170 (carbobenzoxy-valyl-phenylalanial; Z-Val-Phe-CHO) has been shown to impair infection by SARS-CoV-1 and EBOV and is under clinical study for use in COVID-19 [148,151,152], Z LVG CHN2 (N-benzyloxycarbonyl-leucyl-valyl-glycine diazomethylketone) strongly suppresses HSV replication [153] and inhibits the entry step of MERS and SARS-CoV-2 [148,154], and ONO 5334 (N-[(1S)-3-[(2Z)-2-[(4R)-3,4-dimethyl-1,3-thiazolidin-2-ylidene]hydrazinyl]-1-(oxan-4-yl)-2,3-dioxopropyl]cycloheptanecarboxamide) is a cathepsin K inhibitor that impairs the proper processing of SARS-CoV-2 S protein within the endosome, thus inhibiting its fusogenic properties [148].

In addition, the effective and selective inhibitory activity against cathepsin B and/or cathepsin L of a variety of natural products emerged as useful antiviral therapeutics targeting viral entry pathways [155,156,157,158,159]. The most studied commercially available natural cathepsin inhibitor is E-64 (L-trans-Epoxysuccinyl-leucylamido (4-guanidino) butane), isolated from the fungus *Aspergillus japonicus*, which has the advantages of high potency and low toxicity [160]. It has been shown to inhibit the disassembly of reovirus virions after endocytosis [161], prevent upregulation of cathepsins and enhance viral clearance in RSV-infected lungs [118], and block MERS-CoV and SARS-CoV at the entry stage [162]. Other natural cathepsin antagonists include the linear lipopeptide gallinamide A [163], isolated from a *Schizothrix* sp. cyanobacterium, which selectively inhibits cathepsin L, as well as tokaramide A and miraziridine A [164,165], isolated from the marine sponge *Theonella* aff. *mirabilis* and aloperine, a component of the seeds and leaves of *Sophora alopecuroid,* which are selective inhibitors of cathepsin B [156]. In particular, the marine natural product gallinamide A potently inhibits SARS-CoV-2 infection in vitro, with EC_50_ values in the nanomolar range [166]. The quinolizidine-type alkaloid aloperine was shown to inhibit HIV-1 entry into cells by blocking the virus from fusion with the host cell membrane [167], to prevent HCV propagation in primary human hepatocytes and block HCV cell-to-cell viral transmission [168]. Aloperine derivatives were obtained with enhanced antiviral activity towards IAV [169] and high anti-EBOV and anti-Marburg virus activity both in vitro and in vivo [156]. A schematic list of the above described cathepsin inhibitors is reported in Table 2.

Selective cathepsin inhibitors have been extensively used in basic and translational research, allowing a better understanding of the pathogenesis of the infectious diseases caused by viruses and providing valuable information for the development of antiviral drugs and vaccines. It is notable that several cathepsin inhibitors are already successfully employed in clinical practice for the treatment of some viral infections [170].

## 4. Conclusions

Cathepsins are a group of proteolytic enzymes with a broad spectrum of substrates and multiple functions at different locations inside and outside of the cells. In addition to their primary physiological roles in protein turnover and normal cellular metabolism, cathepsins fulfill many additional functions essential for cellular homeostasis: they participate in both the innate and adaptative immune responses, such as antigen presentation and TLR activation, hormone and growth factor processing, autophagy, apoptosis and necroptosis, and inflammation, as well as the processing of transcription factors involved in cell proliferation and differentiation. Altered expression and/or functional profiles of cathepsins have been found in a wide range of pathological states, thus making them potential biomarkers and/or therapeutic targets for many diseases. Here, we have focused on the involvement of cathepsins in the pathogenesis of viral infections. The life cycle of a virus in a host cell includes viral determinants attachment to cell surface factors and/or receptors, entry by either endocytosis or membrane fusion mechanisms, intracellular trafficking, replication and transcription of the viral genome, assembly of newly made virion particles, and egress to infect neighboring cells, thus propagating the infection. In addition, to ensure the replication and spread of the infection through the host organism, pathogenic viruses have developed several strategies to hijack host defenses, including the impairment of immune responses, the manipulation of apoptosis, the modulation of metabolism, modification of the redox environment, and others. Interestingly, robust evidence has demonstrated the involvement of cathepsins in both processes by which viruses infect the host organism and escape the host defenses. In this review, some representative examples of the molecular mechanisms by which cathepsins support the interactions of viruses with target cells at different steps of their life cycle have been reported.

Due to the emergence of infectious diseases that cause pandemics and therefore pose a serious threat to public health and global stability, many research efforts have been focused on the development of effective antiviral drugs. These include several selective cathepsin inhibitors, some of which have been repurposed to combat the new emergence of COVID-19. Thus, here we have also reported some examples and applications of the incredible variety of cathepsin inhibitors developed to date for the treatment of various infectious diseases that represent promising wide-spectrum antiviral agents.

## Figures and Tables

**Table 1:** Cathepsins Involved in Human Viral Infections and Their Mechanism of Action.

Cathepsin	*M*_W_ * (kDa)	Cellular Localization	Enzymatic Activity	Interacting Virus	Mechanism of Action
**Serine cathepsins**					
A	54	Endo/lysosome	Endopeptidase, Carboxypeptidase	SARS-CoV	Antigen processing downregulation [126]
G	29	Endo/lysosome Extracellular space	Endopeptidase	Respiratory syncytial virus	Activation of pattern recognition receptors and immune response hijacking [118]
**Aspartate cathepsins**					
D	45	Endo/lysosome Cytosol Extracellular space	Endopeptidase	Hepatitis B	Autophagy impairment [137]
E	43	Endo/lysosome	Endopeptidase	Respiratory syncytial virus	Activation of pattern recognition receptors and immune response hijacking [118]
**Cysteine cathepsins**					
B	38	Endo/lysosome Cytosol Nucleus Plasma membrane Extracellular space	Endopeptidase, Carboxypeptidase	Ebola	Processing of viral glycoprotein prior to fusion with the cell membrane [63,64,65]
				Human papilloma virus type 16	Binding, internalization and trafficking at the plasma membrane, in the endolysosome, or vesicles [67,69]
				Reoviruses	Disassembly of the viral particles in the late endosomes [70,71,72]
				SARS-CoV and SARS-CoV-2	Activation of S protein for entry by endocytosis [74,75,80,81,85]
				Nipah	Processing viral fusion protein [88]
				Ectromelia	Immune response impairment and replication induction [121]
				Human immunodeficiency virus type 1	Decreased antigen processing and presentation, replication [122]
				Influenza A	Impaired MHC II antigen processing [123]
				Respiratory syncytial virus	Activation of pattern recognition receptors and immune response hijacking [118]
				Noroviruses	Activation of apoptosis and replication induction [138]
				Dengue	Apoptosis activation [139]
				>Coxsackievirus B3	Inflammasome activation, pyroptosis [140]
C	52	Endo/lysosome Cytosol	Endopeptidase	Human immunodeficiency virus type 1	Decreased antigen processing and presentation, replication [122]
				Respiratory syncytial virus	Activation of pattern recognition receptors and immune response hijacking [118]
				Cytomegalovirus	Inhibition of viral replication [143]
H	37	Endo/lysosome Nucleus	Endopeptidase, Aminopeptidase	SARS-CoV	Antigen processing downregulation [126]
				Respiratory syncytial virus	Activation of pattern recognition receptors and immune response hijacking [118]
K	37	Endo/lysosome Nucleus Extracellular space	Endopeptidase	SARS-CoV-2	Protein S processing [85]
				Respiratory syncytial virus	Activation of pattern recognition receptors and immune response hijacking [118]
L	38	Endo/lysosome Cytosol Nucleus Plasma membrane Secretory vesicles Extracellular space	Endopeptidase	Ebola	Processing of viral glycoprotein prior to fusion with the cell membrane [63,64,65,66]
				Human papilloma virus type 16	Binding, internalization and trafficking at the plasma membrane, in the endolysosome, or vesicles [67,69]
				Reoviruses	Disassembly of the viral particles in the late endosomes [70,71,72]
				SARS-CoV, MERS-CoV and SARS-CoV-2	Activation of S protein for entry by either fusion or endocytosis [73,74,75,78,79,80,81,82,83,84,85]; apoptosis activation facilitating viral dissemination [141,142]
				Hendra	Processing of the viral fusion protein [87]
				Herpes Simplex Virus -1 and -2	Heparanase up-regulation, viral egress [89,95,102,103]
				Dengue	Heparanase up-regulation, viral egress [105,106]
				Ectromelia	Immune response escape, replication [121]
				Respiratory syncytial virus	Activation of pattern recognition receptors and immune response hijacking [118]
S	37	Endo/lysosome Cytosol Nucleus Plasma membrane Extracellular space	Endopeptidase	Reoviruses	Disassembly of the viral particles in the late endosomes [71]
				SARS-CoV-2	Protein S processing [85]
				Ectromelia	Immune response escape, replication [121]
				Human immunodeficiency virus type 1	Decreased antigen processing and presentation, replication [122]
				Hepatitis C	Impairment of MHC II maturation [125]
				SARS-CoV	Antigen processing downregulation [126]
				Respiratory syncytial virus	Activation of pattern recognition receptors and immune response hijacking [118]
				Dengue	Activation of apoptosis [139]
V	37	Endo/lysosome Secretory vesicles Extracellular space	Endopeptidase	SARS-CoV-2	Protein S processing [85]
W	42	Endo/lysosome Extracellular space	Endopeptidase	Influenza A	Escape from late endosomes [86]
				Respiratory syncytial virus	Activation of pattern recognition receptors and immune response hijacking [118]
Z	34	Endo/lysosome Cytosol	Endopeptidase	Human immunodeficiency virus type 1	Decreased antigen processing and presentation, replication [122]
				Respiratory syncytial virus	Activation of pattern recognition receptors and immune response hijacking [118]

* Molecular weight.

**Table 2:** Examples of effective cathepsin inhibitors for the treatment of infection diseases caused by viruses.

Target	Inhibitor	Virus(es)	Reference(s)
Cysteine cathepsins	K11777	EBOV, Paramyxoviruses, CoVs	[146]
	Apilimod	SARS-CoV-2	[147,148,149]
Cathepsins B and/or L	MDL 28170	EBOV, SARS-CoV-1, SARS-CoV-2	[148,151,152]
	Z LVG CHN2	HSV MERS and SARS-CoV-2	[153] [148,154]
	Gallinamide A	SARS-CoV-2	[166]
	Aloperine	EBOV HIV-1 HCV IAV	[156] [167] [168] [169]
	E-64	Reoviruses RSV MERS and SARS-CoV-2	[161] [118] [162]
Cathepsin K	ONO 5334	SARS-CoV-2	[148]
